# Reconstruction and analysis of the gene network regulating apoptosis in hepatocellular carcinoma based on scRNA-seq data and the ANDSystem knowledge base

**DOI:** 10.18699/vjgb-25-102

**Published:** 2025-12

**Authors:** A.V. Adamovskaya, I.V. Yatsyk, M.A. Kleshchev, P.S. Demenkov, T.V. Ivanisenko, V.A. Ivanisenko

**Affiliations:** Institute of Cytology and Genetics of the Siberian Branch of the Russian Academy of Sciences, Novosibirsk, Russia Novosibirsk State University, Novosibirsk, Russia; Institute of Cytology and Genetics of the Siberian Branch of the Russian Academy of Sciences, Novosibirsk, Russia Novosibirsk State University, Novosibirsk, Russia; Institute of Cytology and Genetics of the Siberian Branch of the Russian Academy of Sciences, Novosibirsk, Russia Novosibirsk State University, Novosibirsk, Russia; Institute of Cytology and Genetics of the Siberian Branch of the Russian Academy of Sciences, Novosibirsk, Russia Novosibirsk State University, Novosibirsk, Russia; Institute of Cytology and Genetics of the Siberian Branch of the Russian Academy of Sciences, Novosibirsk, Russia Novosibirsk State University, Novosibirsk, Russia; Institute of Cytology and Genetics of the Siberian Branch of the Russian Academy of Sciences, Novosibirsk, RussiaNovosibirsk State University, Novosibirsk, Russia Novosibirsk State University, Novosibirsk, Russia

**Keywords:** hepatocellular carcinoma, single cell transcriptomics, apoptosis, gene networks, cognitive system ANDSystem, гепатоцеллюлярная карцинома, транскриптомика одиночных клеток, апоптоз, генные сети, когнитивная система ANDSystem

## Abstract

Hepatocellular Carcinoma (HCC) is the most common primary liver cancer characterized by rapid progression, high mortality rate and therapy resistance. One of the key areas in studying the molecular mechanisms of HCC development is the analysis of disturbances in apoptosis processes in hepatocytes. Throughout life apoptosis ensures the elimination of old and defective cells while the attenuation of this process serves as one of the leading factors in carcinogenesis. In this study we reconstructed and analyzed the gene network regulating hepatocyte apoptosis in humans based on single-cell transcriptome sequencing (scRNA-seq) data and the ANDSystem knowledge base which employs artificial intelligence and computational systems biology methods. Comparative analysis of gene expression revealed weakened transcription of genes involved in the regulation of inflammatory processes and apoptosis in tumor hepatocytes compared to hepatocytes of normal liver tissue. The reconstructed network included 116 differentially expressed genes annotated in Gene Ontology as genes involved in the apoptotic process (apoptotic process GO:0006915), along with their 116 corresponding protein products. It also included 16 additional proteins that, while lacking GO apoptosis annotation, were differentially expressed in HCC and interacting with genes and proteins participating in the apoptosis process. Computational analysis of the gene network identified several key protein products encoded by the genes NFKB1, MMP9, BCL2, A4, CDKN1A, CDK1, ERBB2, G3P, MCL1, FOXO1. These proteins exhibited both a high degree of connectivity with other network objects and differential expression in HCC. Of particular interest are proteins CDKN1A, ERBB2, IL8, and EGR1, which are not annotated in Gene Ontology as apoptosis participants but have a statistically significant number of interactions with genes involved in apoptosis. This indicates their role in regulating programmed cell death. The obtained results can guide the design of new experiments studying the role of apoptosis in carcinogenesis and aid in the search for novel therapeutic targets and approaches for HCC therapy using apoptosis modulation in malignant hepatocytes. Furthermore, the proposed approach to reconstructing and analyzing the apoptosis regulation gene network in hepatocellular carcinoma can be applied to analyze other tumor forms providing a systemic understanding of disturbances in key regulatory processes in oncogenesis and potential therapy targets

## Abstract (ru)

Гепатоцеллюлярная карцинома – наиболее распространенный первичный рак печени, характеризующийся быстрым прогрессированием, высокой летальностью и устойчивостью к терапии. Одним из ключевых направлений в изучении молекулярных механизмов развития гепатоцеллюлярной карциномы является анализ нарушений процессов апоптоза в гепатоцитах. На протяжении всей жизни благодаря апоптозу происходит элиминация старых и дефектных клеток, тогда как ослабление апоптотической гибели служит одним из ведущих факторов канцерогенеза. В настоящем исследовании выполнены реконструкция и анализ генной сети регуляции апоптоза гепатоцитов у человека на основе данных секвенирования транскриптома одиночных клеток (scRNA-seq) и базы знаний ANDSystem, использующей методы искусственного интеллекта и компьютерной системной биологии. Сравнительный анализ экспрессии генов показал ослабление транскрипции генов, вовлеченных в регуляцию воспалительных процессов и апоптоза, в опухолевых гепатоцитах по сравнению с гепатоцитами нормальной ткани печени. Реконструированная сеть включала 116 дифференциально экспрессирующихся генов, аннотированных в Gene Ontology как гены, вовлеченные в процесс апоптоза (apoptotic process GO:0006915), 116 соответствующих белков, а также 16 дополнительных белков, не имеющих GO-аннотации, но дифференциально экспрессируемых при гепатоцеллюлярной карциноме и вовлеченных во взаимодействия с генами и белками, участвующими в процессе апоптоза. Компьютерный анализ генной сети выявил ряд ключевых белков – продуктов генов NFKB1, MMP9, BCL2, A4, CDN1A, CDK1, ERBB2, G3P, MCL1, FOXO1, демонстрирующих как высокое число связей с другими объектами сети, так и дифференциальную экспрессию при гепатоцеллюлярной карциноме. Особый интерес представляют белки CDKN1A, ERBB2, IL8 и EGR1, не аннотированные в Gene Ontology как участники апоптоза, но обладающие статистически значимым числом взаимодействий с генами, вовлеченными в апоптоз, что указывает на их роль в регуляции программируемой клеточной гибели. Полученные результаты могут найти применение для планирования новых экспериментов по изучению роли апоптоза в канцерогенезе и поиска новых мишеней и подходов для терапии гепатоцеллюлярной карциномы, основанных на модуляции апоптоза в злокачественных гепатоцитах. Предложенный подход к реконструкции и анализу генной сети регуляции апоптоза при гепатоцеллюлярной карциноме может быть использован для анализа других форм опухолей и дает системное представление о нарушениях ключевых регуляторных процессов в онкогенезе и потенциальных мишенях для терапии.

## Introduction

Hepatocellular carcinoma (HCC) is the most common primary liver cancer arising from the malignant transformation of hepatocytes. Approximately 750,000 people die from this disease worldwide each year (Ganesan, Kulik, 2023). This malignancy is characterized by marked resistance to anticancer drugs and a high rate of recurrence (Zou et al., 2025), underscoring the relevance of investigating both the molecular mechanisms of tumorigenesis and the development of tumor resistance – and, on this basis, identifying targets for anticancer therapy. The principal risk factors for HCC include chronic infection with hepatitis B and C viruses, alcoholic cirrhosis, and non-alcoholic steatohepatitis; other established risk factors comprise obesity, type 2 diabetes mellitus, and tobacco smoking (Ogunwobi et al., 2019).

Viral infections and/or adverse environmental factors (exposure to hepatotoxic agents) induce alterations in the functioning of a number of signaling pathways in hepatocytes, leading to their malignant transformation and the development of HCC. It has been established that the hepatitis B virus X protein (HBx) suppresses the activity of the pro-apoptotic protein p53, impairs DNA repair, and activates several signaling cascades (STAT, NF-κB, AP-1, etc.) involved in cell proliferation and survival, thereby promoting HCC progression (Jiang Y. et al., 2019). The pathogenesis of HCC involves changes in: (a) growth factor signaling pathways such as insulin-like growth factor (IGF), epidermal growth factor (EGF), platelet-derived growth factor (PDGF), fibroblast growth factor (FGF), and hepatocyte growth factor (HGF/MET); (b) signaling pathways related to cell differentiation, including WNT, Hedgehog, and Notch; and (c) angiogenesis- related pathways driven by vascular endothelial growth factor (VEGF) and FGF (Dhanasekaran et al., 2016). In addition, disruption of apoptosis – programmed cell death – makes a crucial contribution to HCC progression (Fabregat, 2009). Chronic liver inflammation resulting from hepatitis B or C virus infection or exposure to adverse environmental factors leads to hepatocyte apoptosis accompanied by a compensatory increase in their proliferation, which, under conditions of high oxidative stress caused by inflammation, results in the accumulation of DNA mutations and an increased likelihood of malignant transformation of hepatocytes (Yang et al., 2019). Moreover, apoptosis plays a key role in eliminating malignant cells; therefore, activation of apoptosis is one of the mechanisms of action of anticancer drugs in HCC (Hajizadeh et al., 2023). It has been shown that suppression of the extrinsic and intrinsic apoptosis pathways – particularly by regulatory microRNAs – may be associated with the development of HCC and poor clinical outcomes (Khlebodarova et al., 2023). It has also been established that the hepatitis B virus HBx protein suppresses the activity of the pro-apoptotic protein p53, contributing to the initiation and progression of HCC (Jiang Y. et al., 2019). Available data indicate that disruption of the balance between pro-apoptotic and anti-apoptotic proteins in hepatocytes is one of the factors underlying HCC development and the emergence of drug resistance (Ladd et al., 2024; Wu et al., 2024). This necessitates investigating the mechanisms by which apoptotic pathways in hepatocytes are perturbed during HCC development and identifying key regulatory nodes of apoptosis, the expression of which differs between healthy and tumor hepato- cytes.

It is well known that disturbances in the interactions among tumor cells, the stroma, and immune cells play an important role in disease progression, fostering HCC development, the emergence of drug resistance, and recurrence (Xue et al., 2022). Notably, HCC exhibits a high degree of cellular heterogeneity, which highlights the importance of methods that probe the molecular processes of HCC development at the single-cell level (Li X. et al., 2022).

One such method – single-cell transcriptome sequencing – provides valuable information on gene expression features across different cell types within tumor tissue. This is particularly relevant when comparing malignantly transformed hepatocytes within the tumor to normal hepatocytes from histologically unaltered liver tissue (Zhang et al., 2022). However, differential expression analysis alone is insufficient to elucidate the mechanisms of tumor transformation. Based on such experimental data, it is necessary to reconstruct gene networks – ensembles of coordinately functioning genes – which provide valuable insights into dysregulated molecular mechanisms of gene–gene interactions responsible for the development of pathological processes (Saik et al., 2019; Ivanisenko V.A. et al., 2022; Antropova et al., 2023; Butikova et al., 2025).

The aim of our study was to reconstruct and analyze the gene network regulating apoptosis in hepatocytes in human hepatocellular carcinoma using an integrated approach that combines single-cell transcriptomic data with the ANDSystem software-information platform designed for gene network reconstruction based on automated analysis of scientific publications and biomedical factual databases (Demenkov et al., 2011; Ivanisenko V.A. et al., 2015, 2019). The system employs artificial intelligence methods and an ontological description of the domain, ensuring high coverage and accuracy in knowledge extraction from diverse sources of experimental information (Ivanisenko T.V. et al., 2020, 2022, 2024).

By comparing scRNA-seq transcriptomic data for normal hepatocytes and hepatocytes malignantly transformed in HCC, we identified 1,853 differentially expressed genes (DEGs). Using ANDSystem, we reconstructed an interaction network between the DEGs and genes annotated in Gene Ontology as involved in apoptosis (GO:0006915). Analysis of the resulting gene network highlighted several DEGs, the products of which (including BCL2, NFKB1, FOXO1, MCL1, CDKN1A, ERBB2, IL8, and EGR1) exhibit significant connectivity with components of the apoptosis network. Notably, some of these proteins (CDKN1A, ERBB2, IL8, EGR1) were not annotated in Gene Ontology as apoptosis participants, underscoring their potential novelty and importance for understanding the mechanisms of programmed cell death in HCC. In addition, based on scRNA-seq data, we observed decreased expression of key inhibitors of apoptosis in hepatocellular carcinoma cells. This finding suggests that evasion of apoptosis in HCC may be driven not by the enhancement of anti-apoptotic mechanisms but, on the contrary, by disruption of pro-apoptotic signaling pathways. The results obtained may be useful for planning further experimental studies aimed at elucidating the mechanisms of apoptosis regulation in hepatocytes in HCC and are also of interest for developing targeted therapeutic strategies aimed at modulating apoptotic processes in tumor cells of the liver.

## Materials and methods

**GEO database.** For the analysis, we used single-cell transcriptome sequencing data from primary hepatocellular carcinoma (HCC) specimens and paired histologically normal liver tissues, available in the NCBI Gene Expression Omnibus (GEO) under accession GSE149614. Data from eight patients were analyzed (patients 3, 4, 5, 6, 7, 8, 9, and 10).

**Transcriptome data analysis.** Single-cell RNA-sequencing (scRNA-seq) data processing and downstream analyses were performed in Python using the Scanpy package (v1.9.3) (Wolf et al., 2018). Initial filtering included: (1) removing cells with detected expression for fewer than 100 genes, and (2) removing genes detected in fewer than 3 cells. Normalization was carried out with scanpy.pp.normalize_total(), followed by a log1p transformation. Cell clustering was performed using the Leiden algorithm (Traag et al., 2019). Differentially expressed (marker) genes for each identified cluster were determined with scanpy.tl.rank_genes_groups(), employing the Wilcoxon rank-sum test.

Based on the expression of known hepatocyte marker genes (ALB, HNF4A, SERPINA1, CYP3A4, TAT, TF) (Si- Tayeb et al., 2010) and the clustering results, cells classified as hepatocytes were selected. For subsequent comparative analyses between tumor and normal hepatocytes, pseudobulk samples (Squair et al., 2021) were generated for each patient by aggregating expression values across all cells separately for tumor and normal tissue.

Statistically significant differences in gene expression between the pseudobulk tumor group and the pseudobulk normal hepatocyte group were identified in R using DESeq2 (v1.42.0) (Love et al., 2014). Differentially expressed genes were defined by thresholds of p-value < 0.05 and |logFC| > 0.5.

**Reconstruction of gene networks.** Reconstruction and analysis of the gene network regulating hepatocyte apoptosis in human hepatocellular carcinoma were performed using the ANDSystem software-information platform (Demenkov et al., 2011; Ivanisenko V.A. et al., 2015, 2019). The effectiveness of ANDSystem has been demonstrated in a number of studies, including reconstruction of the endothelial apoptosis regulatory network in lymphedema (Saik et al., 2019) and investigations of molecular mechanisms associated with hepatocellular carcinoma (Demenkov et al., 2023; Khlebodarova et al., 2023). The system has also been applied to the interpretation of omics data – metabolomics (Ivanisenko V.A. et al., 2022, 2024) and proteomics (Momynaliev et al., 2010; Larina et al., 2015) – demonstrating its versatility and applicability to diverse data types and diseases

The network reconstruction comprised several stages. First, using the Query Wizard of the ANDVisio software module (Demenkov et al., 2011), a graphical user interface within ANDSystem, we reconstructed an associative gene network that included genes and their protein products involved in apoptosis. The list of human protein-coding genes participating in apoptosis was obtained from The Gene Ontology Resource (https://geneontology.org/) for the term GO:0006915 “apoptotic process”.

At the second stage, we searched for novel proteins involved in the regulation of apoptosis in hepatocytes during HCC development. We considered as candidates those proteins that are not annotated in The Gene Ontology Resource as apoptosis participants but regulate the expression of the initial genes involved in apoptosis.

To identify such proteins, using the Pathway Wizard in ANDVisio, we retrieved all direct relationships of the types Expression regulation, Expression upregulation, Expression downregulation, and Interaction from the protein products of all DEGs identified in the experiment to the DEGs involved in apoptosis according to Gene Ontology

We then assessed the statistical significance of the specificity of the linkage between the identified proteins and the baseline apoptosis gene network constructed in stage 1. The specificity metric was defined as the proportion of a protein’s interactions that connect to genes in the network relative to the total number of that protein’s genome-wide interactions. The statistical significance of the deviation between the observed number of a given protein’s interactions with network genes and the number expected by chance was evaluated using the hypergeometric distribution:

**Formula. 1.**
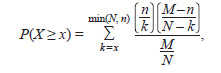
Formula 1

where M is the total number of protein-coding genes in the database, n is the number of genes in the analyzed gene network, N is the total number of human genes that interact with the protein under study, and x is the number of network genes that interact with the protein under study.

P-values were calculated using the Python library (scipy. stats.hypergeom). To correct for multiple testing, the Bonferroni adjustment (Narkevich et al., 2020) was applied, under which DEGs were considered statistically significant if their Bonferroni-adjusted p-value satisfied p < 0.05. All computations were performed using statsmodels and other standard Python tools

Thus, the final gene network regulating apoptosis during HCC development included both the DEGs and their products annotated in Gene Ontology as participating in the apoptotic process, and the protein products of DEGs that were statistically significantly linked to this apoptosis network but not annotated as apoptosis participants in Gene Ontology.

**Gene network analysis.** For each network component (gene or protein), ANDSystem computed the Network Connectivity metric, defined as the number of other network objects (nodes) to which the component is connected (i. e., its degree). Network hubs were defined as proteins and genes, Network Connectivity of which exceeded the critical value (quantile) corresponding to a p-value of 0.05. The quantile was calculated from the empirical distribution of Network Connectivity across all nodes of the gene network. Thus, the number of connections for hub nodes was statistically significant at p < 0.05.

**Phylostratigraphic analysis of gene networks.** The evolutionary age of genes was determined using the GenOrigin database (http://chenzxlab.hzau.edu.cn/) (Tong et al., 2021), which provides gene age annotations across species inferred by phylostratigraphic analysis. To assess the statistical significance of differences in the distribution of gene ages between the full set of human protein-coding genes and the genes in the reconstructed apoptosis network of hepatocytes in HCC, we applied a hypergeometric test. The probability of observing m or more genes from a given age interval among M network genes was calculated using the hypergeom.pmf function from SciPy. The analysis was performed for the 20 age intervals represented in GenOrigin. The following parameters were used in the calculations: N – the total number of human protein-coding genes; n – the number of human protein-coding genes in a given age interval; M – the number of genes in the reconstructed network; m – the number of network genes within the interval under analysis. Differences were considered statistically significant at p < 0.05.

**Functional annotation of gene sets.** Functional annotation of the genes represented in the network was performed using the web-based Database for Annotation, Visualization and Integrated Discovery (DAVID 2021) (https://david. ncifcrf.gov/; Sherman et al., 2022) with default settings. Over-representation analysis of Gene Ontology terms describing biological processes, molecular functions, and cellular components, as well as KEGG pathways (i. e., enrichment analysis of gene sets to identify key biological processes associated with the genes under study), was carried out for (i) the complete set of DEGs identified from the hepatocyte transcriptome analysis and (ii) the subset of DEGs included in the hepatocyte apoptosis regulatory gene network. In DAVID, over-representation of GO terms and KEGG pathways was evaluated using Fisher’s exact test (Sherman et al., 2022). Statistical significance of enrichment was defined as a Bonferroni–Šidák-adjusted p-value < 0.05 (Šidák, 1967).

## Results

**Analysis of differential gene expression in HCC**

As a result of comparing single-cell transcriptomes (malignantly transformed tumor hepatocytes vs. hepatocytes from histologically normal liver tissue), 1,853 differentially expressed genes (DEGs) were identified. The data for these DEGs are provided in Table S11. Among them, 964 genes showed increased expression and 889 genes showed decreased expression in tumor hepatocytes compared with normal liver cells. The results of the functional annotation of DEGs using the DAVID web resource – namely, the lists of significantly overrepresented Gene Ontology terms and KEGG pathways – are presented in Tables S2 and S3. The ten most significant biological process terms (those with the highest proportion of DEGs associated with the term relative to the total number of DEGs) for the upregulated and downregulated gene sets are shown in Table 1.


Supplementary Materials are available in the online version of the paper: https://vavilov.elpub.ru/jour/manager/files/Suppl_Adam_Engl_29_7.xlsx


**Table 1.**
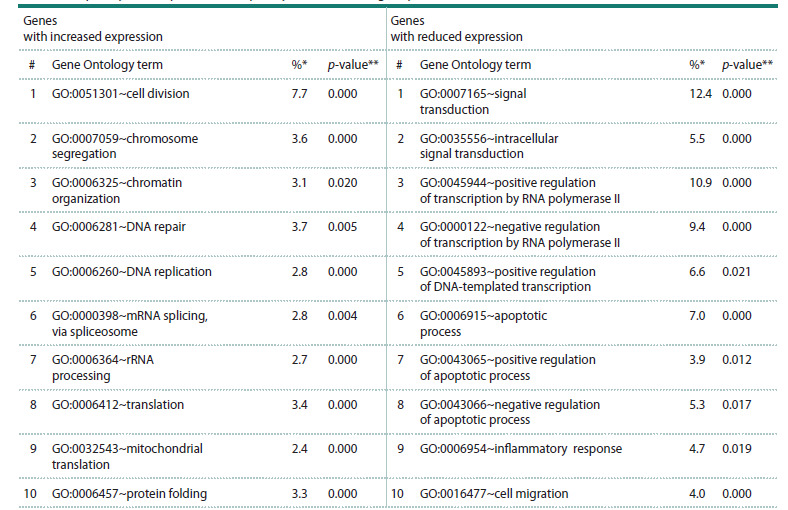
Overrepresented Gene Ontology terms for genes with increased and decreased expression in tumor hepatocytes compared with hepatocytes from histologically normal liver tissue in HCC * Proportion of genes associated with the given term relative to the total number of up- or downregulated genes; ** p-value for the statistical significance of Gene Ontology term over-representation with the Bonferroni–Šidák correction. The table reports the ten most significant terms (those with the highest proportion of DEGs associated with the term relative to the total number of DEGs) describing biological processes for the upregulated and downregulated gene sets.

For the genes with increased expression in malignantly transformed cells, significantly overrepresented terms were related to cell division (#1, #2 in Table 1), chromatin organization (#3 in Table 1), DNA repair and replication (#4, #5 in Table 1), mRNA splicing (#6 in Table 1), rRNA processing (#7 in Table 1), protein translation (#8, #9 in Table 1), and protein folding (#10 in Table 1). For the upregulated genes, KEGG pathways related to oxidative phosphorylation (hsa00190: Oxidative phosphorylation) and DNA replication (hsa03030: DNA replication) were significantly overrepresented (Table S2).

For the genes with decreased expression, significantly overrepresented terms described intracellular signal transduction (#1, #2 in Table 1), transcriptional regulation (#3–5 in Table 1), positive and negative regulation of apoptosis (#6–8 in Table 1), inflammation (#9 in Table 1), cell migration (#10 in Table 1), T-cell receptor signaling pathways (#10 in Table S3), and receptor tyrosine kinases (#11 in Table S3).

For the genes with increased expression, significantly overrepresented KEGG pathways included the MAPK signaling pathway (hsa04010), NF-κB signaling (hsa04064), chemokine signaling (hsa04062), and T-cell receptor signaling (hsa04660) (Table S3).

**Gene network of DEGs involved in the apoptosis process according to Gene Ontology data**

As described in the “Materials and methods” section, reconstruction of the gene network regulating apoptosis in hepatocytes during HCC development was carried out in two stages. Given the well-established importance of apoptosis in HCC (Hajizadeh et al., 2023; Ladd et al., 2024; Wu et al., 2024), as well as the over-representation of apoptosis-related processes among downregulated genes identified in our study (Table 1) in malignantly transformed hepatocytes, the first stage incorporated into the gene network those genes and their protein products that, according to Gene Ontology, are involved in apoptosis and the expression of which in tumor hepatocytes differs from that in hepatocytes from histologically normal liver tissue. Of the 746 protein-coding genes (Table S4) annotated in The Gene Ontology Resource under the term “apoptotic process” (GO:0006915), 116 (16 % of all genes annotated to this term) were differentially expressed in malignantly transformed hepatocytes. Of these, 49 genes were upregulated and 67 genes were downregulated in tumor hepatocytes compared with healthy liver cells, accounting for 42.2 and 57.8 %, respectively, of the 116 apoptosis-related DEGs. The associative gene network reconstructed using ANDSystem (Fig. S1) comprised the 116 DEGs involved in apoptosis and their 116 protein products. Characteristics of this network are presented in Table 2 (column “Gene network, stage 1”); its visualization is shown in Fig. S1, and the full list of components (proteins and genes) is provided in Table S5.

**Table 2.**
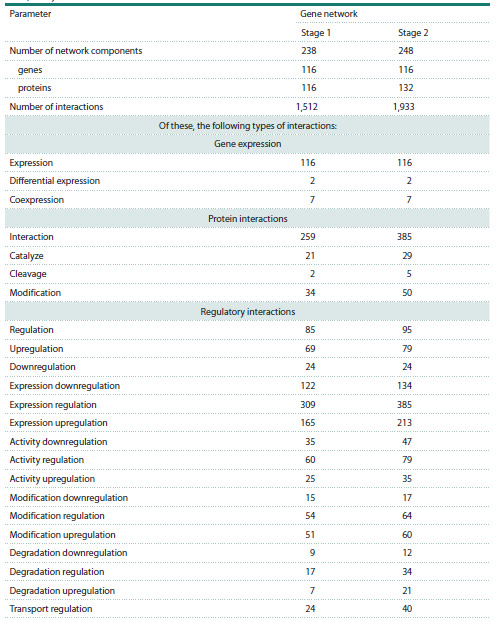
Characteristics of associative networks of genes and proteins involved in apoptosis of hepatocytes in HCC

At the second stage, to identify novel protein regulators of apoptosis during the malignant transformation of hepatocytes, the network reconstructed in stage one was expanded by adding the protein products of all DEGs revealed by the comparative analysis of transcriptomes from malignantly transformed hepatocytes and hepatocytes of histologically normal liver tissue. In expanding the network, we selected relationship types pertaining to gene expression regulation – expression regulation, expression upregulation, expression downregulation, and interaction. We found that, of the 116 apoptosis-related DEGs, the expression of 68 genes (59 %) is regulated by 223 proteins encoded by genes that are differentially expressed in tumor hepatocytes relative to normal liver tissue, but are not annotated in Gene Ontology as participating in apoptosis. The list of these genes is provided in Table S6. Of them, 102 genes were upregulated and 121 genes were downregulated.

According to functional annotation, the downregulated genes were significantly overrepresented (Bonferroni-adjusted -value < 0.05) for biological processes including leukocyte cell–cell adhesion (GO:0007159), neutrophil chemotaxis (GO:0030593), cell division (GO:0051301), and positive regulation of the PI3K/Akt signaling pathway (GO:0051897).

Next, for the 223 candidate proteins potentially involved in regulating hepatocyte apoptosis during HCC development, we assessed the statistical significance of their specificity of association with the apoptosis regulatory gene network. For each protein, we calculated the probability that the observed fraction of its interactions with network genes relative to its total interactions with human protein-coding genes could arise by chance. As a result, 16 DEGs (11 downregulated and 5 upregulated) were identified as significantly associated (Bonferroni-adjusted p-value < 0.05) with 43 apoptosis genes (Table 3). As seen in Table 3, the products of IL8, ERBB2, EGR1, TGFB2, and CDKN1A have the highest numbers of links to DEGs already annotated in Gene Ontology as apoptosis participants. Proteins encoded by CDN1A, ETS2, EGR1, BACH2, KLF5, and FEN1 are transcription factors according to The Human Transcription Factors database (Lambert et al., 2018; https://humantfs.ccbr.utoronto.ca/).List of proteins encoded by DEGs of malignantly transformed hepatocytes that are involved in the regulation of apoptosis in HCC but are not annotated in Gene Ontology as participants in apoptosis (GO:0006915, apoptotic process)

**Table 3.**

List of proteins encoded by DEGs of malignantly transformed hepatocytes that are involved in the regulation of apoptosis in HCC but are not annotated in Gene Ontology as participants in apoptosis (GO:0006915, apoptotic process) Note. Number of interactions to apoptosis DEGs – the number of expression-regulatory links from the protein to genes involved in apoptosis according to Gene Ontology; Total number of links – the number of links from the protein to all components of the final gene network (genes and proteins); Expression – direction of the gene’s expression change in tumor hepatocytes relative to normal cells (increased; decreased); p-value – statistical significance of the protein’s association with apoptosis genes, computed using the hypergeometric test with the Bonferroni correction. Proteins are sorted in descending order of the significance of their association with the apoptosis network. Transcription factors are shown in bold, according to The Human Transcription Factors database (Lambert et al., 2018; https://humantfs.ccbr.utoronto.ca/).

The final gene network of hepatocyte apoptosis in HCC is shown in Fig. S2, and its characteristics are presented in Table 2 (column “Gene network, stage 2”). The complete list of proteins and genes in the network is provided in Table S7. As seen in Table 2, upon expanding the initial apoptosis gene network with proteins that regulate the expression of apoptosis genes, the number of links of all types increased, with the exception of downregulation. Network hubs – that is, the nodes (genes or proteins), Network Connectivity (the number of other nodes connected to a given node) of which exceeded the critical (quantile) threshold corresponding to a p-value of 0.05 (see “Materials and methods”) – are listed in Table 4.

**Table 4.**
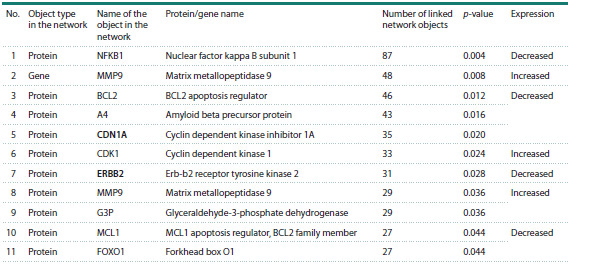
Hubs of the apoptosis gene network in hepatocytes in human hepatocellular carcinoma Note. p-value – the critical threshold (quantile) calculated from the observed distribution of Network Connectivity across all nodes of the gene network. Proteins not previously annotated in Gene Ontology as participants in the apoptotic process are shown in bold..

A total of 11 network hubs were identified (Table 4), 10 of which are proteins, and one is the gene MMP9, the product of which also appears among the network hubs. According to scRNA-seq data (Table S1), the expression of genes encoding three proteins (CDK1, MMP9, G3P) was increased in malignantly transformed hepatocytes compared with hepatocytes from histologically normal liver tissue, whereas the expression of genes encoding the remaining seven proteins (NFKB1, BCL2, A4, CDKN1A, ERBB2, MCL1, FOXO1) was decreased. The genes encoding two network hubs – CDKN1A and ERBB2 – had not previously been annotated in Gene Ontology as participants in the apoptotic process.

**Network of gene expression regulation involved in hepatocyte apoptosis during the development of hepatocellular carcinoma**

**Fig. 1.**
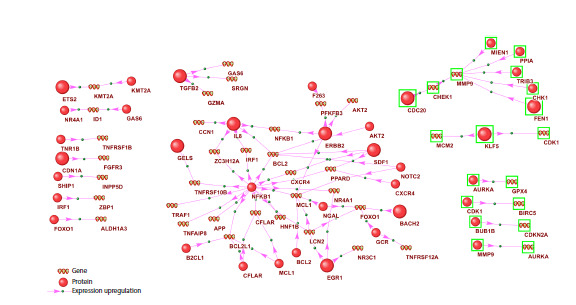
Gene network of expression activation for gene components of the apoptosis regulatory network during HCC development Proteins and genes with increased expression are outlined in green; those with decreased expression are not outlined. Proteins that had not previously been annotated in Gene Ontology as participants in apoptosis are shown as larger circles. Shown are only the protein components of the hepatocyte apoptosis regulatory network in HCC (see Fig. S2) that activate (type of interaction – expression upregulation) the expression of gene components of the same network.

**Fig. 2.**
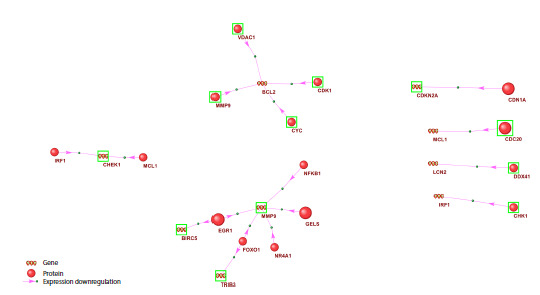
Gene network of expression repression for gene components of the apoptosis regulatory network during HCC development Proteins and genes with increased expression are outlined in green; those with decreased expression are not outlined. Proteins not previously annotated in Gene Ontology as participants in apoptosis are shown as larger circles. Shown are only the protein components of the hepatocyte apoptosis regulatory network in HCC (see Fig. S2) that suppress (type of interaction – expression downregulation) the expression of gene components of the same network

Taking into account the scRNA-seq-identified changes in the expression of genes, the products of which are involved in hepatocyte apoptosis during HCC development, we analyzed gene expression regulation within the final apoptosis network. To this end, we filtered the edges of the reconstructed network, retaining only those proteins that either enhance (edge type “expression upregulation,” Fig. 1) or suppress (edge type “expression downregulation,” Fig. 2) the expression of genes comprising the final apoptosis regulatory network.

The expression-activation network (Fig. 1) comprised 38 proteins that activate the expression of 40 gene components of the apoptosis network. According to ANDSystem, NFKB1 activates the expression of 15 genes (including BCL2, MCL1, CFLAR, etc.), IL-8 activates 5 genes, ERBB2 activates 4 genes, and EGR1, SDF1, and TGFB2 each activate 3 genes; the remaining proteins in the expressionactivation network regulate fewer than three apoptotic genes. In our scRNA-seq analysis (Table S1), both these regulators and their target genes exhibited decreased expression in malignantly transformed hepatocytes compared with hepatocytes from histologically normal liver tissue. By contrast, the matrix metalloproteinase gene MMP9, which was upregulated, is activated, according to ANDSystem, by five proteins (MEIN1, PPIA, TRIB3, CHK1, FEN1), the expression of which was also increased in tumor hepatocytes. In addition, CDC20, FEN1, KLF5, and their target genes showed increased expression

The expression-repression network (Fig. 2) of genes involved in apoptosis in HCC comprised 15 proteins connected by “expression downregulation” type of interactions to 9 genes. According to ANDSystem, the expression of MMP9 can be suppressed by five proteins (NFKB1, GELS, NR4A1, FOXO1, EGR1), the expression of which is reduced in malignantly transformed hepatocytes according to scRNA-seq, which may account for the elevated MMP9 expression observed in the scRNA-seq analysis. The expression of BCL2, which is decreased in tumor hepatocytes, can be suppressed by four proteins (CDK1, VDAC1, MMP9, CYC), the expression of which is increased in malignant hepatocytes compared with hepatocytes from healthy liver tissue. Among the proteins involved in apoptosis regulation in HCC but not annotated in Gene Ontology as participants in this process, the expression-repression network included EGR1, CDN1A, GELS, and CDC20.

**Phylostratigraphic analysis of the gene network**

**Fig. 3.**
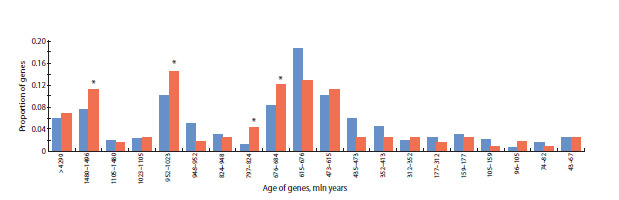
Distribution of the evolutionary age of genes in the reconstructed hepatocyte apoptosis network during HCC development The X-axis shows gene age intervals (million years) according to the GenOrigin database; the Y-axis shows the proportion of genes in each interval. Blue bars indicate the distribution for the full set of human protein-coding genes; red bars indicate the distribution for genes in the reconstructed hepatocyte apoptosis network in HCC. * – denotes statistical significance of the difference in gene representation for a given age interval between the full set of human protein-coding genes and the reconstructed network.

The analysis of the evolutionary age distribution of genes in the reconstructed apoptosis network in HCC is presented in Figure 3. The proportion of genes in the reconstructed apoptosis network was significantly higher ( p < 0.05, hypergeometric test) than that among all human protein-coding genes in the following age intervals: (1) 1,480–1,496 million years, 13 genes; (2) 952–1,023 million years, 17 genes; (3) 797–824 million years, 5 genes; (4) 676–684 million years, 14 genes.

## Discussion

Apoptosis is a tightly regulated and evolutionarily conserved program of cell death that performs key functions in normal physiological processes such as embryogenesis and tissue homeostasis in the adult organism. Resistance to apoptosis is a well-known hallmark of cancer cells that supports their survival and tumor growth (Kashyap et al., 2021). However, the literature also reports that apoptotic processes can be activated in tumor cells, especially at late stages of neoplasm development. Thus, although evasion of apoptosis is a wellestablished oncogenic mechanism (Moyer et al., 2025), tumor cell populations cannot continuously suppress the apoptotic program across all cells within a tumor (reviewed in Morana et al., 2022). This indicates specific features of apoptosis regulation during malignant progression that depend on tumor stage, tissue of origin, and cell type, given the wellknown cellular heterogeneity of tumors (Li C. et al., 2020). Therefore, detailed investigation of the molecular genetic mechanisms of apoptosis in different types of malignancies – particularly HCC – at the single-cell level is required.

In the present study, using publicly available scRNA-seq data, we performed a comparative analysis of the transcriptomes of malignantly transformed hepatocytes and hepatocytes from histologically normal liver tissue, and we reconstructed the gene network regulating apoptosis in hepatocytes during human hepatocellular carcinoma. Analysis of the scRNA-seq data and gene expression regulation within the reconstructed network showed that expression of genes NFKB1, BCL2, and MCL1 – network hubs (Table 4) – is reduced in malignant hepatocytes compared with healthy cells. The BCL2 and MCL1 proteins are known key inhibitors of apoptosis, as they prevent activation of BAX/BAK, which is required to increase mitochondrial membrane permeability and subsequently activate effector caspases (Newton et al., 2024). Upregulation of BCL2 expression is considered one of the major mechanisms by which cells acquire resistance to apoptosis during malignant transformation (Moyer et al., 2025). However, in our study we observed decreased expression of BCL2 and MCL1 in HCC hepatocytes, which – according to analysis of the apoptosis regulatory network – may be due both to reduced expression of proteins that activate BCL2 and MCL1 expression (such as NF-κB, SDF1, ERBB, IL-8; Fig. 1) and to increased expression of proteins that suppress BCL2 expression (Fig. 2).

It is noteworthy that NFKB1 is the principal hub of the hepatocyte apoptosis network in HCC (Table 4) and a key protein in the network that activates expression of genes involved in hepatocyte apoptosis (Fig. 2), which, according to ANDSystem, can activate a number of anti-apoptotic genes, including BCL2 and MCL1. In tumors, activation of the NF-κB signaling pathway promotes survival by inhibiting apoptosis (Gupta et al., 2023); therefore, the decreased NFKB1 expression found in our study (Tables S1 and 4) may plausibly increase hepatocyte susceptibility to apoptosis. On the other hand, activation of NFKB1 is reported to be necessary for apoptosis via the extrinsic pathway induced by chemokines – particularly IL1b (Wang P. et al., 2023) – and mediated by the TNFR1 receptor (Moyer et al., 2025). Thus, reduced NFKB1 expression in malignantly transformed hepatocytes could, on the one hand, facilitate apoptosis of malignant hepatocytes by weakening expression of apoptosis inhibitors, but on the other hand hinder induction of extrinsic apoptosis, which requires NF-κB activation. In addition, our scRNA-seq analysis (Table S1) showed increased expression of genes encoding pro-apoptotic proteins in tumor hepatocytes, such as BID – a BAX/BAK activator (Moyer et al., 2025) – and FADD (FAS-associated death domain protein), a key component of the extrinsic apoptotic pathway (Nagata et al., 2017; Kashyap et al., 2021). One of the apoptosis network hubs, cyclin-dependent kinase 1 (CDK1), also shows increased gene expression in malignant hepatocytes (Table S1). G. Massacci et al. (2023) demonstrated that CDK1 phosphorylates BCL2L1, BCL2, and MCL1, thereby suppressing their anti-apoptotic functions. However, that study also emphasized that the role of CDK1 in apoptosis regulation may depend on experimental context and cellspecific features.

Overall, the scRNA-seq data indicate decreased expression of key anti-apoptotic genes and increased expression of important pro-apoptotic genes in malignant hepatocytes compared with healthy hepatocytes. Our results suggest that, in the context of HCC, a reduction in anti-apoptotic protein levels is insufficient to trigger apoptosis. This, in turn, suggests that evasion of apoptosis by upregulating inhibitors of apoptosis is not the predominant mechanism of HCC progression, which may instead be driven by other causes likely related to the hepatocyte microenvironment – particularly dysregulation of inflammatory processes – as supported by scRNA-seq studies (Lu et al., 2022; Jiang S. et al., 2024). We also believe that activating pro-apoptotic effectors, such as caspases, should be a key therapeutic objective

It is well known that NF-κB proteins are major regulators of inflammation, and increased expression stimulates the inflammatory response (Wang P. et al., 2023). Therefore, the reduced expression of the NFKB1 gene, which encodes one member of this family, NFKB1, is consistent with the attenuated expression of genes involved in the inflammatory response in malignant hepatocytes, as indicated by the functional annotation of DEGs (Table 1).

A search for regulatory links between the DEGs controlling hepatocyte apoptosis in HCC and proteins – the products of other DEGs identified by scRNA-seq – allowed us to identify more than 200 proteins (Table S6) that could potentially modulate the expression of genes governing hepatocyte apoptosis during HCC, even though they are not annotated in Gene Ontology as regulators of this process. Notably, functional annotation of the genes encoding these proteins revealed in tumor cells a reduced expression of genes, the products of which support leukocyte migration and adhesion – chemokines (CCL5, CXCL2, CXCL8, CXCL1), transforming growth factor-β2 (TGFB2), the tyrosine kinase SYK, and integrin ITGA4. However, according to ANDSystem, these same proteins can regulate key nodes of the hepatocyte apoptosis regulatory network. In particular, CCL5 induces expression of matrix metalloproteinase 9 (MMP9) (Sevenich, Joyce, 2014), which is one of the principal hubs of the reconstructed apoptosis regulatory network in HCC hepatocytes. MMP9 is a member of the multifunctional family of zinc-dependent endopeptidases and is activated during inflammation and in certain cancers. Matrix metalloproteinases cleave extracellular matrix proteins and play crucial roles in cellular apoptosis, angiogenesis, tumor growth, and metastasis (Verma et al., 2015). MMP9 is known to be capable of inducing apoptosis (Liang et al., 2019). These findings indicate that reduced expression of genes encoding key immune defense components may promote tumor progression not only by weakening the immune response to transformed cells but also by influencing apoptotic processes within them.

At the same time, the previously proposed statistical approach (Yatsyk et al., 2025) for assessing the significance of a given protein’s or gene’s association with a network of interest (in this case, apoptosis), together with analysis of the reconstructed network, enabled us to prioritize several proteins – potential participants in the regulation of the apoptotic process in hepatocytes – the altered expression of which is likely to disrupt apoptosis regulation in hepatocytes and thereby contribute to the onset and progression of HCC. These proteins (ERBB2, CDN1A, IL8, EGR1) are significantly associated with the hepatocyte apoptosis regulatory network in HCC and act as central regulators (hubs) influencing a large number (>20) of its nodes.

The ERBB family of erythroblastic leukemia viral oncogene homologs, which includes the epidermal growth factor receptor (EGFR) and ERBB2, ERBB3, and ERBB4, regulates a broad range of essential cellular functions, such as survival, growth, and migration of tumor cells, and has therefore attracted attention as a therapeutic target in cancer (Chen et al., 2024). ERBB2, a member of this family, the expression of which was reduced in malignant hepatocytes according to scRNA-seq, has not previously been annotated as involved in apoptosis regulation, yet it emerged as a statistically significant hub of the reconstructed apoptosis regulatory network (Table 4). Elevated ERBB2 expression is associated with breast tumor growth, and suppression of ERBB2 and ERBB3 induces apoptosis in breast cancer cells (Xiang et al., 2010). Although there are no data on the role of ERBB2 in apoptosis induction in HCC, our network analysis indicates that this protein regulates several apoptosis-related proteins and genes in HCC, including NFKB1, AKT2, CDK1, MCL1, and FOXO1. In particular, ERBB2 has been shown to phosphorylate cyclin-dependent kinase CDK1, increasing the resistance of cancer cells to apoptosis induced by the cytostatic anticancer drug paclitaxel (Vahedi et al., 2015). ERBB2 also appears to activate expression of the anti-apoptotic genes NFKB1, AKT2, and MCL1 (Fig. 1), which are downregulated in malignant hepatocytes according to our scRNA-seq data. Thus, ERBB2 is an important potential node in the regulation of apoptosis in hepatocytes, and changes in its expression may contribute to HCC development

IL-8, also known as CXCL8, is a pro-inflammatory chemokine of the CXC family. Elevated IL-8 levels are associated with poor prognosis across various cancers, including hepatocellular carcinoma. In HCC, increased IL-8 expression is also linked to enhanced metastatic potential of tumor cells (Han et al., 2023). Choi et al. (2016) showed that IL-8 knockdown promoted apoptosis in HCC cells.

CDN1A (also known as CDKN1A), cyclin-dependent kinase inhibitor 1A encoded by the CDKN1A gene, has not previously been annotated in Gene Ontology as a protein involved in apoptosis; however, its role in apoptosis during HCC development has been discussed in the literature (Thanga- velu et al., 2024). Reports emphasize that the role of CDN1A in regulating apoptosis during tumorigenesis is contextdependent, as CDKN1A can both suppress and promote apoptosis (Manu et al., 2019). Experimental data indicate that CDKN1A is a p53 target and can stimulate apoptosis in tumor cells by activating the TNF receptor or the proapoptotic protein BAX, or by modulating the intrinsic apoptotic pathway via changes in mitochondrial membrane permeability (Abbas, Dutta, 2009). The natural compound N-trans-feruloyloctopamine can enhance apoptosis of HCC cells through its interaction with CDKN1A (Ma et al., 2021).

ANDSystem data indicate that this protein is one of the central nodes of the apoptosis regulatory network in hepatocytes during HCC development. It interacts with other network hubs, in particular with well-known apoptosis regulators such as NFKB1, BCL2, and CDK1. However, scRNA-seq analysis showed that CDKN1A expression was reduced in tumor hepatocytes compared with normal liver cells (Table 4). These findings suggest that attenuation of CDKN1A expression in hepatocytes may represent an important link in HCC pathogenesis, facilitating tumor-cell evasion of apoptosis; nevertheless, its role in hepatocyte apoptosis regulation in HCC requires further experimental investigation

Early growth response protein 1 (EGR1) suppresses proliferation and enhances apoptosis of malignantly transformed cells in many tissues and organs, including the liver (reviewed in Wang B. et al., 2021). It has also been shown that EGR1 can inhibit HCC growth by repressing transcription of PFKL (phosphofructokinase-1, liver type) and by inhibiting aerobic glycolysis in tumor cells (Pan et al., 2024). In our study, EGR1, the expression of which is reduced, acts as an activator of genes (LCN2, NR3C1, NR4A1; Fig. 1) involved in apoptosis control, the expression of which is likewise reduced in malignant hepatocytes. Our results suggest that decreased EGR1 expression may be one of the mechanisms underlying weakened apoptosis during malignant transformation

The use of phylostratigraphic analysis to assess gene evolutionary age is important for studying the evolution of gene networks and identifying their key components (Mustafin et al., 2021). Notably, most genes in the hepatocyte apoptosis network and those in the overrepresented age intervals are older than 600 million years (Fig. 3), whereas relatively young genes are scarce, indicating evolutionary conservation of the network genes and their importance for cellular viability. In particular, the overrepresented group of genes aged 1,480–1,496 million years corresponds to the period of mitochondrial–eukaryotic cell symbiosis (Raval et al., 2023). During these stages of symbiosis, many genes responsible for mitochondrial programmed cell death evolved, including key factors regulating cytochrome c release and oxidative stress control – early adaptations that maintained symbiotic balance (Zmasek, Godzik, 2013). Moreover, we found a statistically significant excess of genes in the hepatocyte apoptosis network, relative to the human genome as a whole, within the 952–1,023-million-year interval. This interval includes, in particular, proteins such as BCL2 – a network hub – and BCL2L1. These proteins are well-known key inhibitors of apoptosis (Moyer et al., 2025). Orthologs of BCL2 family genes are found in sponges (Porifera), placozoans (Placozoa), and hydras (Hydra) (Banjara et al., 2020), i. e., at a relatively early stage of metazoan evolution. The critical role of apoptosis in innate and adaptive immunity suggests that this function arose early in the evolution of multicellularity and likely preceded the adaptation of apoptosis to other processes – such as development, homeostasis, and removal of damaged cells in Metazoa – laying the groundwork for complex multicellular life (Suraweera et al., 2022). Thus, changes in hepatocyte gene expression during HCC involve highly conserved genes – including the network hub BCL2 – that, beyond apoptosis, may regulate other cellular processes, underscoring the complexity of regulatory interactions during malignant transformation.

Accordingly, our study – using an integrated approach that included hepatocyte transcriptome analysis and reconstruction/ analysis of a DEG network involved in apoptosis – provides new insights into the regulation of hepatocyte apoptosis during human HCC development. Our findings, which show decreased expression of key apoptosis inhibitor genes, support the view that evasion of apoptosis is not invariably characteristic of cancer cells and that the role of apoptosis in tumor development depends on the cell type, tissue context, and tumor microenvironment (Morana et al., 2022). In addition, reduced expression in malignant hepatocytes of genes involved in inflammatory control, together with decreased NFKB1 – a central regulator of inflammation (Wang P. et al., 2023) – points to an important role for interactions between hepatocytes and the immune system in HCC development, warranting further experimental and theoretical investigation. The identified network hubs (NFKB1, MMP9, BCL2, A4, CDN1A, CDK1, ERBB2, G3P, MCL1, FOXO1) may serve as useful targets for modulating apoptosis in hepatocytes in HCC therapy, an increasingly promising direction (Ladd et al., 2024; Wu et al., 2024).

## Conclusion

Analysis of scRNA-seq data from normal and malignantly transformed hepatocytes revealed changes in the expression of genes involved in the control of hepatocyte apoptosis in HCC. In malignant hepatocytes, expression of the key apoptosis inhibitors BCL2 and MCL1 was decreased, as was the expression of genes involved in the inflammatory response. These findings indicate that evasion of apoptosis by upregulating key apoptosis inhibitors does not appear to be a characteristic feature of hepatocytes during HCC development. Reconstruction and analysis of the hepatocyte apoptosis – regulatory network in HCC showed that reduced expression of NFKB1 may be an important factor underlying the decreased expression of a range of apoptosis-related genes, including BCL2 and MCL1. In addition, network reconstruction and analysis identified several key genes (NFKB1, MMP9, BCL2, A4, CDN1A, CDK1, ERBB2, G3P, MCL1, FOXO1) that both display differential expression in malignant versus healthy hepatocytes and function as hubs of the hepatocyte apoptosis network in HCC. Dysregulated expression of these genes may lead to apoptosis dysregulation in tumor cells.

Among the DEGs, we also identified genes (CDKN1A, ERBB2, IL8, EGR1) that, although not annotated in Gene Ontology as apoptosis participants, exhibited numbers of regulatory interactions of their products with apoptosis genes that significantly exceeded chance expectations according to a hypergeometric test. This suggests that the proteins encoded by these genes play specific roles in regulating hepatocyte apoptosis in HCC and represent promising candidates for further investigation.

The results obtained can be used to guide future experimental studies on the regulation of hepatocyte apoptosis in HCC. The hypotheses proposed may facilitate the development of targeted therapeutic strategies aimed at modulating programmed cell death in malignant liver cells.

## Conflict of interest

The authors declare no conflict of interest.
